# Comparing gene annotation enrichment tools for functional modeling of agricultural microarray data

**DOI:** 10.1186/1471-2105-10-S11-S9

**Published:** 2009-10-08

**Authors:** Bart HJ van den Berg, Chamali Thanthiriwatte, Prashanti Manda, Susan M Bridges

**Affiliations:** 1 Department of Basic Sciences, College of Veterinary Medicine, Mississippi State University, Starkville, MS 39762, USA; 2 Department of Computer Science and Engineering, Box 9637, Mississippi State University, Starkville, MS 39762, USA; 3 Institute for Digital Biology, Mississippi State University, Starkville, MS 39762, USA

## Abstract

The widespread availability of microarray technology has driven functional genomics to the forefront as scientists seek to draw meaningful biological conclusions from their microarray results. Gene annotation enrichment analysis is a functional analysis technique that has gained widespread attention and for which many tools have been developed. Unfortunately, most of these tools have limited support for agricultural species. Here, we evaluate and compare four publicly available computational tools (Onto-Express, EasyGO, GOstat, and DAVID) that support analysis of gene expression datasets in agricultural species. We use AgBase as the functional annotation reference for agricultural species. The selected tools were evaluated based on i) available features, usage and accessibility, ii) implemented statistical computational methods, and iii) annotation and enrichment performance analysis. Annotation was assessed using a randomly selected test gene annotation set and an experimental differentially expressed gene-set – both from chicken. The experimental set was also used to evaluate identification of enriched functional groups.

Comparison of the tools shows that they produce different sets of annotations for the two datasets and different functional groups for the experimental dataset. While DAVID, GOstat and Onto-Express annotate comparable numbers of genes, DAVID provides by far the most annotations per gene. However, many of DAVID's annotations appear to be redundant or are at very high levels in the GO hierarchy. The GOSlim distribution of annotations shows that GOstat, Onto-Express and EasyGO provide similar GO distributions to those found in AgBase while annotations from DAVID show a different GOSlim distribution, again probably due to duplication and many non-specific terms. No consistent trends were found in results of GO term over/under representation analysis applied to the experimental data using different tools. While GOstat, David and Onto-Express could retrieve some significantly enriched terms, EasyGO did not show any significantly enriched terms. There was little agreement about the enriched terms identified by the tools.

**Conclusion:**

Different tools for functionally annotating gene sets and identifying significantly enriched GO categories differ widely in their results when applied to a test annotation gene set and an experimental dataset from chicken. These results emphasize the need for care when interpreting the results of such analysis and the lack of standardization of approaches.

## Background

Systems biology research aims to characterize cellular networks and mechanisms by integrating high-throughput "-omics" data from genomics, proteomics, transcriptomics, and metabolomics experiments. It is humanly impossible to manage, analyze and interpret these massive datasets manually. Therefore researchers have developed a wide array of computational tools over the last decade to assist researchers in deriving biological value from the generated data [1,2]. Gene annotation enrichment analysis is a widely used approach, where the over or under-representation of gene ontology (GO) terms in a set of genes is determined statistically. Available tools perform a number of similar functions and each also presents its own unique features. However, the majority of currently available computational tools target well-studied model organisms such as human, mouse, rat and *Arabidopsis*. There are very few publicly available computational tools that include equally important but less studied organisms such as agricultural species. In addition, most tools are only compatible with popular commercial arrays (e.g. Affymetrix and Agilent), while other valuable, widely-used custom arrays are disregarded. This multitude of available tools makes it difficult to the researcher to choose the right tools for the job. Recently, an extensive comparison and summary of 68 gene annotation enrichment analysis tools was published [1], categorizing tools into three classes based on their underlying algorithms. This comparison provides the user with a clear overview of the current availability and differences of a multitude of gene annotation enrichment analysis tools. However, the summary does not provide a side-by-side performance comparison of the tools when applied to biological datasets. The tool features and underlying algorithm(s) do not necessarily reflect the value and functionality of a tool. Our goal is to use an empirical evaluation to provide insight into the obstacles and issues encountered in analysis of gene annotation enrichment, especially when using data generated from agricultural species.

Here, we evaluate and compare four gene annotation enrichment analysis tools: Onto-Express [3], EasyGO [4], GOstat [5], and DAVID [6]. All are categorized by Huang et. al. as Class 1 singular enrichment analysis (SEA) tools [1]. Although Huang et al. describe 44 available Class 1 SEA tools, we selected only the tools that directly support chicken gene input for this study. Gene Set Enrichment Analysis (GSEA) tools such as GenePattern [7] were not selected because they do not directly support chicken gene identifiers. In addition to the four selected SEA tools, the AgBase [8] database is used as a baseline for functional annotation of agricultural species. Since Gene Ontology (GO) annotation is the *de facto *method for functional annotation [9], we have chosen tools that primarily use GO as their annotation resource in gene annotation enrichment analysis, although some of the tools also have other biological databases integrated (e.g. KEGG, REACTOME). However, the standard vocabulary provided by GO allows easy comparison of the results produced by different tools.

We used a test annotation gene set of 60 randomly selected chicken genes (Test Set) with identifiers compatible with all of the tools to evaluate the gene annotation capabilities of each tool. In addition, we use an experimental dataset of differentially expressed genes identified from a FHCRC 13 k chicken cDNA microarray (Experimental Set) to evaluate the gene annotation and gene annotation enrichment analysis performance of each tool when applied to a real-life dataset. Comparison of gene annotation enrichment analysis tools is quite challenging due to the differences in the underlying algorithms, databases, multiple correction methods, output formats, and many other features of the tools. We defined a standard set of tool parameters (Table 1) with the goal of generating comparable gene annotation enrichment results. We also provide researchers with a general summary of challenges, obstacles and possible solutions when dealing with gene annotation enrichment analysis tools applied to agricultural species.

**Table 1:** Standard set of tool parameters

Parameter	Value
Maximum p value	0.10
Maximum GO depth	5
False discovery correction	FDR
Statistical method	OntoExpress & EasyGO: Hypergeometric GOstat & DAVID: Fisher's Exact

A consistent set of tool parameters was used where possible to make the results more comparable. Note that there was no one set of statistical methods available for all tools.

## Results and discussion

### Data set generation

The online Molecular Biology Database Collection lists a total of 1170 databases publicly available online [10]. Many of these tools generate their own identifiers making it extremely difficult for researchers to retrieve information from public databases with compatible identifiers. The tools used here integrate several publicly available databases, each with their own identifier compatibility. In order to use all of the tools, we had to convert our EST probe identifiers to at least two other identifiers compatible with the tools. Our Test Set includes randomly selected genes for which we were able to find compatible identifiers for almost all genes for the four tools (Additional file 1). However, converting the large list of experimental EST probe identifiers to other identifiers resulted in a reduced number of identifiers as shown in Table 2. This is primarily due to the mapping of multiple ESTs to a single gene. In addition, some ESTs did not map to known genes. This becomes important when assigning functional information to EST probes, as redundancy of genes, proteins and their related GO terms could bias the statistically significant biological theme of the dataset.

**Table 2:** Identifier mapping for experimental data set

Identifier	FHCRC whole array	FHCRC differentially expressed
Probe ID	15227	53
Entrez Gene ID	9277	33
UniprotKB accession no.	8838	33

A variety of gene identifiers are accepted as input by the evaluated tools. Entrez Gene IDs and UniProtKB accession numbers corresponding to each Array ID were retrieved to make the data sets compatible with each tool. Not all EST sequences on the microarrays have corresponding identifiers in all databases.

### Tool feature evaluation

Computational tools are often designed to accomplish a specific goal and then expanded with additional features. Changing statistical methods and needs of researchers combined with continual generation of new data makes maintenance and regular updating of existing tools essential. We compared feature similarities and differences for the selected tools (Additional file 2). Huang *et al. *have previously provided a summary of tool features [1] of the underlying statistical methods and annotation visualization methods of a wide range of tools, but provided only a brief description of the annotation database and the species' compatibility of a few tools. For the tools used in this comparison, we present an expanded discussion of species compatibility and databases used and also discuss several other practical features influencing the usability of the tools.

The core of each tool is its underlying database. Several tools have multiple bio-databases implemented for information retrieval. All the tools support GO modelling, while DAVID and Onto-Express also incorporate other bio-databases (e.g. KEGG, REACTOME). As mentioned earlier, maintenance and updating is essential for a tool, especially for their underlying database(s). We found that database update intervals for the evaluated tools range from weekly to annually. Comparing update schedules of several major repository databases (RefSeq [11], Genbank [12], UniProtKB/SwissProt [13], GOA [14], IPI [15]) we suggest that a scheduled monthly database update would be a minimum to provide the researcher with the latest annotation information. The ability to upload custom annotations into the gene annotation enrichment analysis or the database provides a short-cut to overcome out-dated or incomplete annotation information. The tools evaluated here offer either direct custom annotation upload or upload upon request.

Adequate user-support for a tool is essential to enable users to access its full range of tool capabilities and to use the tool efficiently and effectively. All of the tools we evaluated provide a description of the tool, a user's manual, and sometimes additional educational resources. DAVID provides a helpful wizard-style guide through the analysis, which makes the upload and analysis of datasets simple and rapid.

Result storage on the tool's server for future access supports the researcher's ability to rapidly access previous results without having to re-analyze entire datasets. EasyGO provides a session ID valid for two weeks to retrieve results, whereas GOstat provides a session ID for 24-hours, but also provides an offline result-viewer for researchers to download. DAVID and Onto-Express do not provide data storage.

The annotation evidence code describes the type of evidence used to assign a GO annotation to a gene product (e.g. inferred from direct assay, inferred from genetic interaction or inferred by electronic annotation) and is a reflection of the strength of the evidence supporting the annotation. Recently, a method for evidence code-based Gene Annotation Quality (GAQ) analysis was published [16]. This method calculates a GAQ score that allows researchers to quantitatively assess the quality of the functional annotations assigned to their data set and is currently available upon request at the AgBase database [17]. AgBase is the only annotation resource in this study that provides the annotation evidence code directly in the annotation result export and thus supports GAQ score calculation.

All tools provide researchers the option of using a default or a custom uploaded background gene dataset for gene annotation enrichment analysis. This allows researchers to calculate the true statistical enrichment significance when using microarray data. In microarray analysis, the number of genes that one is able to detect is limited to what is on the slide. When using the entire genome as background, the statistically significant enrichment is biased since more genes are considered than actually can experimentally be detected. Uploading a custom background (i.e. all genes on the microarray) allows the researcher to eliminate this statistical bias.

DAVID is the only tool in this study that presents only over-represented functional terms. This has the potential to bias the biological conclusion, since under-represented terms also provide valuable information for understanding the biological processes at work. For example, when comparing control and disease datasets, the lack of expression of a certain gene or functional category may be a signature for the disease.

### Implemented statistical methods for determining GO term enrichment

The underlying statistical methods implemented in a tool contribute not only to the applicability of the tool to datasets, but also allows researchers the freedom to choose the statistical method(s) they deem suitable for their data. The methodology behind the statistical approaches has previously been extensively described and discussed [1,18-20]. The tools included in this study implement a diverse set of statistical methods for determining GO term enrichment as shown in Table 3. In addition to the statistical methods available for calculating over/under representation, each tool also provides multiple testing correction methods as shown in Table 4. Onto-Express, EasyGO, GOstat and DAVID provide the False Discovery Rate (FDR) by Benjamini [21] and/or by Yekutieli [22]. In addition, GOstat provides a Holm p-value correction and DAVID provides a Bonferroni correction. The Bonferroni correction is the most stringent of all false detection correction methods and could lead to a substantial loss of data (false negatives). The Benjamini FDR is most popular because it does not assume independence of genes. Tian et. al. [23] discusses short-comings of commonly used statistical approaches that assume independence among genes. This assumption clearly does not hold in biological systems and Tian *et al. *describe an alternative statistical method for determining statistical differential ontology. The public availability of this method, however, is not clear. Lewin et al. [24] described a similar statistical problem and have implemented their solution in the tool FatiGO [25]. The distinction between the statistical approaches has been described by Huang et al [1] and categorized into classes based on the gene annotation enrichment analysis approach.

**Table 3:** Statistical tests implemented in evaluated tools

Tool	Chi-Square	Hypergeometric	Fisher's Exact	Binomial
Onto-Express	√	√	√	√
EasyGO	√	√		√
GOstat	√		√	
DAVID			√ *	

The subset of tools selected provides a wide variety of statistical tests for the significance of gene annotation enrichment analysis.

* Modified Fisher's exact test known as EASE.

**Table 4:** Multiple testing correction methods implemented in evaluated tools

Tool	Benjamini FDR	Yekutieli FDR	Holm p-value	Bonferroni	Sidak
Onto-Express	√		√	√	√
EasyGO		√			
GOstat	√	√	√		
DAVID	√	√		√	

Multiple testing correction is used to correct for the occurrence of false positive identifications by adjusting p-values derived from multiple statistical tests.

Another point of interest on which most biologists concur is that the arbitrary selection of a statistical significance "cut-off" will often result in a loss of legitimate biological information. Therefore, researchers need to remember that these computational tools are intended to be evaluative and not definitive to the biology. They provide a starting place for hypothesis generation and testing.

### GO annotation modelling

AgBase provides researchers with highly curated GO annotations for agricultural species to be used for downstream modelling. The AgBase biocurators provides a preponderance of the GO annotations for the Gene Ontology Annotation for chicken at EBI. Therefore, AgBase is used as a baseline reference for the retrieval of GO terms.

#### Test gene annotation set

The value of a tool lies predominantly in the available functional information in the underlying database. We used a set of 60 randomly selected chicken genes (Test Set) to assess the number of annotations each tool is able to assign. Table 5A shows that all tools recognize all genes in the input except EasyGO. Also, both the number of genes that have annotations assigned and the total number of annotations assigned differ substantially among the tools. This appears to be mainly due to the version of the database used. All the tools rely mainly on importing the GOA database and this import may be out of date with some tools. DAVID however, showed an unusually high number of annotations. DAVID integrates multiple databases (see Additional file 2), that may cause redundancy in GO terms. For example, when searching the UniProtKB accession Q5ZHQ6 in AgBase, GOstat, DAVID and the actual GOA database, we found 6, 14, 15 and 6 GO terms assigned by each resource, respectively. AgBase and UniProtKB shows the same GO terms retrieved, whereas GOstat and DAVID retrieved the same and additional GO terms. Those additional GO terms stem from redundant parent GO terms. For example, for cellular component, five GO terms are assigned by DAVID and GOstat. However, examination of these terms shows that cell (GO:0005623) is a parent of cell part (GO:0044464), which is a parent of membrane (GO:0016020) which is a parent of membrane part (GO:0044425), which is a parent of intrinsic to membrane (GO:0031224). This creates a bias in subsequent gene annotation enrichment analysis, since the same annotations for a particular gene are counted as individual terms, while in reality they are different level descriptions of the same annotation. This could explain the higher number of the annotations retrieved by DAVID.

**Table 5:** Annotation performance

Tool	# Genes input	#Genes recognized	#Genes annotated	#Annotations retrieved
***A. Test gene annotation set***				

Onto-Express	60	60	56	313
EasyGO	60	56	45	339
GOstat	60	60	56	303
DAVID	60	60	58	1662
AgBase	60	60	49	474

***B. Experimental chicken gene set***				

Onto-Express	31	29	24	328
EasyGO	31	31	21	104
GOstat	31	31	25	227
DAVID	31	26	26	615
AgBase	31	27	22	136

For each tool, the number of gene identifiers used as input, the number of genes recognized, the number of genes for which some GO annotation was retrieved, and the total number of annotations for all genes is given for both the Test Set and the Experimental Set.

AgBase retrieves more annotations for the test set than do EasyGO, GOstat and Onto-Express. This could be explained by the manual curation by which AgBase assigns protein annotations that are included into their database. These curated annotations have been submitted to UniProtKB/Swiss-Prot and are awaiting inclusion into the UniProtKB database.

**Figure 1:**
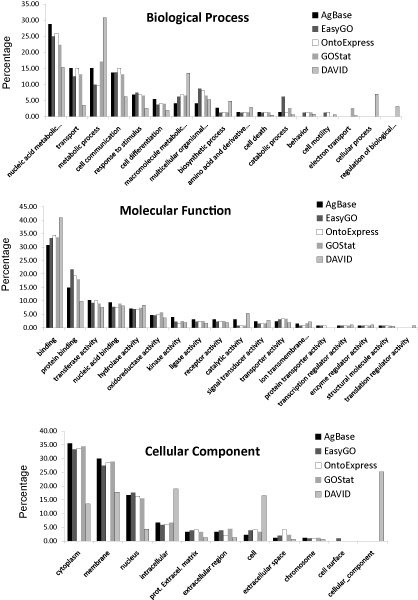
**Comparison GOSlim distribution for the Test Set**. The distribution of the Gene Ontology annotations in the Test Set in different GOSlim categories was computed for the three GO ontologies: Biological Process (BP), Molecular Function (MF) and Cellular Component (CC) using GOSlimViewer at AgBase. AgBase serves as a baseline of retrieved annotations.

Figure 1 compares the GOSlim distribution per ontology of major GO terms of the retrieved annotations for each tool. AgBase was used as the reference annotation resource, since it provides the most recent, highly curated annotations for agricultural species. Based on the annotations retrieved using the Test Set (see Table 6), EasyGO, GOstat and Onto-Express followed a similar representation of major GO terms as AgBase for each ontology. Small percentage differences are present that reflect the underlying version of the annotation database. Interestingly, DAVID shows numerous outliers in "Biological Process" (BP) and "Cellular Component" (CC). Since DAVID retrieved a substantially higher number of annotations for the Test Set (see table 5), the majority of GO annotations are grouped to higher order terms that are so high in the GO tree that they become less informative. This reduced biological detail does not facilitate in-depth modelling of the dataset.

**Table 6:** Gene annotation enrichment analysis

	Experimental Set		
**Ontology**	**BP**	**MF**	**CC**

OntoExpress	81	19	6
EasyGO	1	1	1
GOstat	0	5	-1*
DAVID	33	38	8

The number of GO terms in the Experimental Set found to be enriched for each ontology (Biological process = BP, molecular function = MF, Cellular Component = CC) are given when using the parameters listed in Table 1.

*under-represented GO term

#### Experimental gene set GO-based modeling

**Figure 2:**
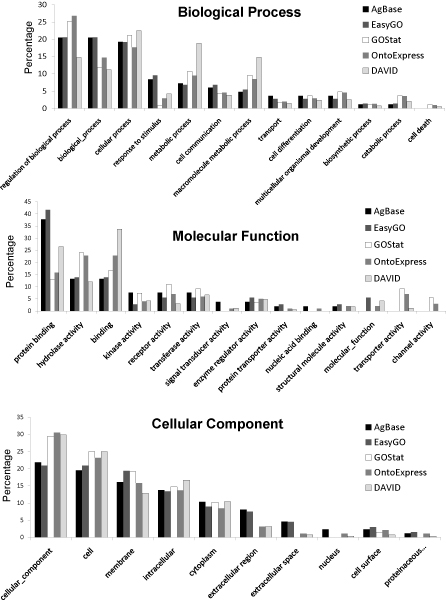
**Comparison GOSlim distribution for the Experimental Set**. The distribution of the Gene Ontology annotations in the Experimental Set in different GOSlim categories was computed for the three GO ontologies: Biological Process (BP), Molecular Function (MF) and Cellular Component (CC) using GOSlimViewer at AgBase. AgBase serves as a baseline of retrieved annotations.

We have compared the results using our experimental set of differentially expressed genes (Experimental Set) in the same way as for the Test Set. Table 5B summarizes the annotation performance for each tool for this data set. Differences are observed not only in gene/identifier recognition, but also in the number of gene annotations assigned by each tool. Excluding the unusual high number of retrieved annotations by DAVID, AgBase and EasyGO retrieved the most annotations for the Test Set, yet retrieve the least annotations for the Experimental Set. DAVID again retrieves the most annotations, but, as discussed earlier, this appears to be due to a great deal of repetition and the inclusion of many very general parent terms. When comparing the GOSlim distribution for the Experimental Set (Figure 2), we can clearly see that the difference in annotation retrieval influences the biological theme of the dataset. For the biological process, all evaluated tools have annotations representing two very general GO slim groups (e.g. "biological_process", "cellular_process"). AgBase and EasyGO show a theme more focused on cell signalling and communication (e.g. "response to stimulus", "cell communication"), whereas DAVID, Onto-Express and GOstat retrieved more annotations to metabolism-related processes (e.g. "metabolic process", "macromolecule metabolic process", "catabolic process"). In terms of molecular function, all evaluated tools again represent global GO terms such as "binding" and "protein binding". But interestingly, GOstat, DAVID and Onto-Express provide additional detailed functions such as "transporter activity" and "channel activity". The cellular component ontology distribution shows an overall similar distribution; however, GOstat does not retrieve annotations for "extracellular region", "extracellular space", and "nucleus" and "proteinacious extracellular matrix". This is interesting since all tools predominantly use the GOA database as their GO annotation resource.

Overall, based on these results, having one dataset and multiple tools could provide different biological conclusions. Researchers need to keep their overall research goal in mind to validate the retrieved annotations and derive conclusions based on an evaluative assumption rather than a conclusive statement.

### Gene annotation enrichment performance

Each evaluated tool is designed to perform functional enrichment analysis on a gene set. While there are multiple accepted statistical methods available, each has their limitations. As described previously [1,2,23] researchers need to decide which methods would be most appropriate for their research model. A comparison of functional enrichment analysis results generated by the evaluated tools provides insight into the performance of each tool. We used the Experimental Set with each tool to generate functional enrichment results. Because there was no one statistical test implemented by all tools (see Table 3), we chose to use statistical tests implemented by at least two tools. Therefore, we compared Onto-Express with EasyGO, because they both implement a hypergeometric statistical method and DAVID with GOstat because they both provide a Fisher's exact test. DAVID uses a modified Fisher's exact test, called EASE, so comparison with GOstat is not conclusive.

Table 6 shows the GO terms that were found significantly enriched (FDR p-value 0.1, GO term depth 5) in the Experimental Set. Both Onto-Express and DAVID found many enriched terms whereas GOstat and EasyGO found only a small number of enriched terms. GOstat is the only tool that does report an under-represented GO terms for Cellular Component.

To gain a better understanding of the biological meaning of the enriched GO terms, we compared the GOSlim distributions for the significantly enriched genes found by each tool. Additional file 3 lists the enriched GO terms retrieved by all tools. The functional enrichment results from the Experimental Set show interesting GO term distributions. For the biological process ontology, GOstat did not find any GO terms represented. EasyGO, DAVID and Onto-Express are in agreement that "response to stimulus" is one of the major GO terms represented. However, additional GO terms from DAVID represent an immunological trend, while Onto-Express find GO terms enriched to a developmental and metabolical trend. The cellular component ontology also shows disagreement where GOstat reports an "intracellular" trend, DAVID an extracellular trend, EasyGO and Onto-Express represent a more global cell location. The molecular function ontology GO terms find agreement by each tool, in that "protein binding" is the major biological trend. Onto-Express find additional details to enzyme activities, while DAVID shows chemokine and cytokine activities.

Although the tools show some agreement for the Experimental Set, there are also substantial differences. This makes it hard to identify a specific biological theme represented in a given dataset. As mentioned earlier, each tool should be considered evaluative and not conclusive in terms of the gene annotation enrichment results and the related biological trends. This comparison demonstrates that even if a dataset is evaluated by multiple tools, it may be difficult to find a general trend that will help the researcher focus on more specific genes of interest.

## Conclusion

No standard GO annotation assignment method has been established in the scientific world. Each tool has advantages and disadvantages in the features it supports and the statistical methods it uses. Having more databases incorporated in a tool does not necessarily positively affect the number of gene annotations retrieved. Gene/protein identifiers play a critical role in database compatibility and annotations retrieved. Availability of GO annotation evidence code would offers a more valuable quantitative assessment (i.e. GAQ score) of assigned annotation quality in the entire dataset. Researchers in the agricultural community would benefit greatly from inclusion of their species in tools such as GenePattern [7] that implement more sophisticated statistical tests and use different analysis techniques.

## Methods

### Test gene dataset

We selected 60 probes from all the structurally annotated probes on the widely used Fred Hutchinson Cancer Research Center (FHCRC) 13 K chicken cDNA microarray (GEO accession GPL2863) [26] to serve as our test gene annotation set (Test Set). Since each tool accepts different gene identifiers, we selected the 60 probes for which we could retrieved the corresponding Entrez Gene ID and UniProtKB accessions via the UniGene database [27] and IPI database [22] (Additional File 1). This set serves as equal input for each tool and is used to evaluate the annotation performance.

### Experimental gene dataset

For the Experimental Set, we used the custom-made FHCRC 13 K chicken cDNA microarray (containing 13,007 features) to represent a real experimental dataset. We used a differentially expressed gene-set, which is previously published; Zhou and Lamont described 53 significantly differentially expressed ESTs using the FHCRC 13 K [28]. As for the Test Set, we retrieved all possible corresponding Entrez Gene IDs and UniProtKB accessions for each probe. Since multiple ESTs can be assigned to one gene, we removed duplicate genes. In addition, some ESTs may not be structurally annotated. Therefore, from the 53 ESTs, we were able to obtain 31 genes for input into each evaluated tool.

### Tool evaluation

The tools used in this comparative study are Onto-Express [3], EasyGO [4], GOstat [5], and DAVID [6]. These tools were chosen because they fulfilled the criteria of being i) operational and freely accessible online; ii) compatible with agricultural species (e.g. chicken, corn, cow) and iii) supportive of GO-based gene annotation enrichment analysis. We also used GOretriever from AgBase [8] to retrieve all possible GO annotations for our datasets. AgBase currently provides the most comprehensive and recent GO annotations for a majority of agricultural species. This allows us to obtain a core reference set of GO annotations for our experimental dataset.

We evaluated each tool via published literature describing the tool and accessed the tool's website for additional information and available features. We evaluated the tools based on i) available features, usage and accessibility; ii) implemented statistical computational methods; iii) annotation performance analysis. The approach for the latter is described in more detail below.

### Computational analysis

We accessed each tool online and submitted each differential expressed data set as input for each tool. Some tools allow users to upload their own background list of genes to calculate enrichment against. We analyzed our Experimental Set with the parameters listed in Table 1. We analyzed the enrichment using common statistical methods available in the tools when possible.

### Performance analysis

We analyzed the results of each tool based on the number of genes recognized, the total number of genes annotated, and the total number of GO annotations found. We compared the over and under representation of GO terms as calculated by each tool and used GOSlimViewer from AgBase [8] and the "GOA and whole proteome GOSlim set" to compare the distribution of the major GO groups represented for each tool's generated dataset.

## Competing interests

The authors declare that they have no competing interests.

## Authors' contributions

BVDB assisted in the projects design, generated the input data-sets, analyzed the results of each tool, and wrote the manuscript draft. CT and PM both generated the results with each tool and evaluated each tool's features. SMB assisted in the project design, progress management and manuscript review.

## Supplementary Material

Additional file 1 **Test gene-set**. Column 1 contains 150 randomly selected EST Array IDs from the FHCRC 13 K chicken cDNA microarray. Column 2 contains all 150 corresponding Entrez Gene IDs and column 3 contains all 150 corresponding UniProtKB accession numbers. The first 60 IDs were used to evaluate the level of annotation provided by each tool. Click here for file

Additional file 2 **Tool Features**. This file contains a summary of key features of the tools and resources evaluated here. Click here for file

Additional file 3 **Gene annotation enrichment GOSlims**. This file contains the GOSlim distribution for each GO ontology category represented by the results of the gene annotation enrichment analysis from the Experimental Set. Click here for file
